# Very early reduction in efficacy of botulinum toxin therapy for cervical dystonia in patients with subsequent secondary treatment failure: a retrospective analysis

**DOI:** 10.1007/s00702-013-1127-5

**Published:** 2013-12-06

**Authors:** Harald Hefter, Constanze Spiess, Dietmar Rosenthal

**Affiliations:** Department of Neurology, University of Düsseldorf, Moorenstraße 5, 40225 Düsseldorf, Germany

**Keywords:** Cervical dystonia, Botulinum neurotoxin, Secondary treatment failure, Neutralizing antibodies, Prevalence of therapy failure, Long-term treatment

## Abstract

The objective of this study was to estimate the probability of development of partial secondary treatment failure (PSTF) in patients with cervical dystonia (CD) who had been treated over up to 9 years with repetitive intramuscular injections of botulinum neurotoxin type A (BoNT/A). The temporal course of treatment response in patients in whom PSTF was detected retrospectively was compared to patients with a normal clinical response. For this purpose, charts of all CD patients treated in our outpatient clinic between 1988 and 2001 were retrospectively analyzed. Extracted data included time of all injections, dose per visit, disease severity measured by TSUI scores, and time of determination of neutralizing antibodies. Final data analysis using a special formal definition of PSTF was based on charts of 568 patients having exclusively been treated with abobotulinumtoxinA. PSTF onset was observed in our CD cohort during the entire treatment period analyzed, with no clustering at any time point. Probability to develop PSTF was 14.5 % in 9 years. Thus, mean PSTF incidence was 1.6 % per year. The mean TSUI score of patients with retrospectively defined PSTF (*n* = 33) became already significantly worse after the second injection when compared with the group without PSTF (*n* = 535). Our data indicate that clinical response in patients developing PSTF later on differs from that of patients without PSTF already very early in the course of botulinum neurotoxin type A treatment, and that PSTF remains undetected at this early stage. Reduced response may therefore be present in a number of CD patients who think they still respond normally to continuous BoNT/A treatment.

## Introduction

Administration of botulinum neurotoxin type A (BoNT/A) has proven safe and efficient in the treatment of cervical dystonia (CD; Simpson et al. 2008) but intramuscular injections of the BoNT protein complex have to be performed repeatedly to continuously suppress muscular hyperactivity (Moore and Naumann 2003). This might result in the formation of neutralizing antibodies (NABs) and increases the risk of secondary therapy failure (STF; Benecke 2012). High doses and booster injections are risk factors for NAB development and STF (Greene et al. 1994; Kessler et al. 1999).

Frequency estimates of NAB formation in CD patients vary considerably from 1.2 % (Brin et al. 2008) using the mouse lethality assay to 40 % (Kranz et al. 2008) including patients with “borderline” antibody values in the mouse hemidiaphragm test, low antibody titers and continued clinical responsiveness. Rates of full STF and partial STF (PSTF) seem to lie in between these extremes and cluster around 3–5 % (Zuber et al. 1993; Kessler et al. 1999; Dressler and Hallett 2006; Mohammadi et al. 2009). Nevertheless, several publications state that NABs can be detected in only about 50 % of the patients developing STF or PSTF (Kessler et al. 1999; Mohammadi et al. 2009; Lange et al. 2009). Obviously, reported rates on STF or NABs are rather confusing. To determine a reliable estimate of incidence and prevalence of PSTF in a cohort of long-term treated CD patients, the following study was designed.

The first sign of STF development is usually a reduction in efficacy duration reported by the patient (Dressler 2004). The treating physician may react by performing clinical screening tests (Kessler and Benecke 1997; Hanna et al. 1999; Birklein and Erbguth 2000; Nestor and Ablon 2011). If these clinical tests support suspected PSTF, serum samples may be screened or directly tested for antibodies against the botulinum neurotoxin with ELISA or Western blot assays (Moore and Naumann 2003; Hanna and Jankovic 1998; Hanna et al. 1999), the mouse hemidiaphragm assay (MHDA; Göschel et al. 1997) or the mouse lethality assay (MLA; Hanna and Jankovic 1998). Therefore, PSTF and/or NABs are only detected when patient and treating physician realize that the current therapy has become insufficient. This unsystematic approach implies that PSTF and NABs remain undetected in a variety of patients resulting in an underestimation of PSTF and/or NAB prevalence.

By means of a more systematic approach, where a small but representative sample of the patient cohort is clinically tested and receives NAB analysis by means of the MHDA, Kranz et al. (2008) estimated NAB prevalence in their CD cohort as up to 40 % including patients with continued responsiveness (see above). Because this method heavily relies on the selection procedure for this small representative patient sample, it may overestimate PSFT or NAB prevalence.

The exact method to estimate PSTF and NAB incidence and prevalence is the Kaplan–Meier analysis of a systematically tested patient cohort. However, this method was not used in recent studies (Brin et al. 2008; Comella et al. 2011; Naumann et al. 2013).

Some reports have suggested that STF occurs early in the course of treatment (Duane et al. 1995; Dressler et al. 2000; Dressler 2004; Lange et al. 2009) with the implication that if PSTF does not occur during the first 2–3 years of treatment it will unlikely occur during subsequent years. This statement is, however, not based on systematic testing of PSTF or NAB occurrence but on the above mentioned clinical approach of testing for NAB presence only if treatment failure becomes obvious. The suggestion that STF develops only at an early treatment stage might have caused patients and physicians to monitor the treatment effects of BoNT injections more carefully at start of therapy than after 8–10 successful injections.

We have therefore screened all charts of CD patients having started BoNT treatment in our outpatient clinic between 1988 and 2001 for the development of PSTF during the course of BoNT therapy to precisely determine PSTF incidence and prevalence in our cohort. We hypothesize that the efficacy of BoNT injections is reduced very early in the treatment of patients with subsequent PSTF compared to patients who do not develop PSTF.

## Patients and methods

### Definition of partial secondary treatment failure

In our outpatient clinic, the TSUI score (Tsui et al. 1986) is the standard tool for monitoring treatment efficacy in CD. Patients usually receive BoNT/A injections every 10–14 weeks and the TSUI score is determined routinely just before reinjection when the effect of the previous injection is already decreasing or has ceased. Partial secondary treatment failure (PSTF) is assumed in a patient when (1) he/she had previously had a good treatment response (when the difference between the TSUI baseline score determined at injection session 1 just before the first injection and the TSUI score determined at injection session number *x* (*x* ≥ 2) is ≥3 points, and (2) a patient’s TSUI score systematically worsens in spite of maintenance or increase of dose starting at injection session number *y* (*y* > *x*) during BoNT/A treatment, and (3) the patient had reported that the last two injections had been less effective than the previous ones. A systematic worsening of the TSUI score was defined as an increase in TSUI scores for more than two points over two treatment cycles (=three consecutive TSUI scores). This definition of PSTF is based on at least four TSUI scores being determined during treatment with at least three consecutive BoNT/A injections.

### Patients

A total of 1,438 charts of patients receiving treatment in our botulinum toxin outpatient clinic between 1988 and 2001 were available; the 704 charts containing data of patients with idiopathic CD were screened further by one of the authors. Only patients exclusively treated with abobotulinumtoxinA (Dysport^®^, Ipsen Ltd.) were included (*n* = 660). Patients missing relevant demographic data (17 patients) or who had been treated with onabotulinumtoxinA (Botox^®^, Allergan Inc.; 17 patients) or incobotulinumtoxinA (NT201 = Xeomin^®^, Merz Pharmaceuticals; 10 patients) were excluded. A further 92 patients were excluded who had received only three BoNT/A injections to avoid a possible placebo effect (ratings too positive after the first injection). The remaining 568 charts of patients with at least four consecutive well-documented abobotulinumtoxinA injections (=treatment over 1 year) were screened for the presence of PSTF according to the definition presented above using the TSUI scores in the charts. Total dose per visit, patient’s report on efficacy of the preceding injection, and date of collecting a serum sample for NAB assays were extracted from the charts. According to the charts, antibody testing had been performed if a patient had reported an insufficient treatment effect and the treating physician suspected NAB induction. Blood samples had been analyzed by means of the mouse hemidiaphragm assay (MHDA; Göschel et al. 1997). The entire cohort was divided into patients who developed PSTF according to the retrospective classification (PSTF subgroup) and patients who did not (NSTF subgroup).

### Statistical analysis

**Fig. 1:**
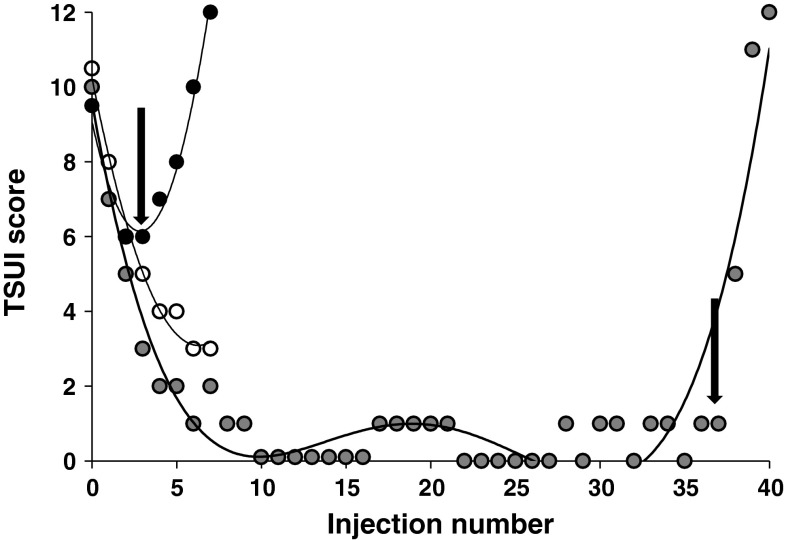
Comparison of the course of BoNT treatment in three patients with an initial TSUI score of 10. Patient 1 (*open circle*) showed continuous improvement over seven treatment cycles. Due to occupational reasons he ceased attending our clinic and his data were censored in the Kaplan–Meier analysis after seven injections. Patient 2 (*dark circle*) initially showed a good response which was reduced after the second injection (indicated by the *arrow* on the *left*). Patient 3 (*gray circle*) who had a better initial treatment response than patients 1 and 2 developed partial secondary treatment failure after 38 treatment cycles (indicated by the *arrow* on the *right*)

The probability of continuous BoNT/A treatment without development of PSTF was estimated using a Kaplan–Meier analysis. The Kaplan–Meier approach takes into account that duration of treatment is patient-dependent. Patient data were censored from further analysis when therapy was interrupted for at least one treatment cycle (implying that the patient had not been treated for half a year). An “event” (=occurrence of PSTF) was defined to have happened at the time when the TSUI score started to worsen systematically (see arrows in Fig. 1). The Kaplan–Meier analysis was not extended beyond 9 years (108 months), because the number of patients (*n* = 230 at 108 months) rapidly decreased after 9 years. The straightforward reason is that BoNT treatment in our clinic was started in 1988 and the number of patients being treated rapidly increased during the first years.

TSUI scores of different injection time points were compared non-parametrically by Wilcoxon rank sum test between the PSTF and the NSTF subgroup. For the sake of comparability and statistics, individual TSUI scores were standardized (for each patient) as percentage of the corresponding baseline TSUI score. Mean TSUI scores and standard deviations were presented as percentages of mean baseline scores (Fig. 3; mean baseline scores for the PSTF and the NSTF group are given in Table 1).

**Table 1:** Baseline characteristics of the partial non-responders (PSTF subgroup) and the remaining study population (NSTF subgroup)

Characteristic	PSTF subgroup	NSTF subgroup
Number of patients	33	535
Female	20 (60.6 %)	302 (56.4 %)
Male	13 (39.4 %)	233 (43.6 %)
Mean age at onset of CD (years)	48.8 ± 12.4	49.7 ± 12.6
Mean TSUI score	9.82 ± 2.01	9.88 ± 2.13

### Approval of the local ethics committee

Data collection was performed retrospectively in 2001 as part of the doctoral thesis of one of the authors (Spiess 2005). We have a recent general approval of the local ethics committee allowing us to take blood samples and publish anonymized clinical data and results of antibody testing of patients having given informed consent.

## Results

Demographic characteristics and TSUI baseline scores of the study population are summarized in Table 1. The stratification according to development of PSTF shows that baseline data were comparable between the PSTF and the NSTF subgroups. One has to keep in mind that the classification whether a patient belonged to the PSTF or the NSTF subgroup was made many years (>11 years) after the TSUI scores were determined. At the time of treatment, the treating physician suspected PSTF (which was clearly different from the definition of PSTF in the present paper) only in 16 patients in whom NABs were tested and were positive.

According to our definition of PSTF, 33 patients (5.8 %) were retrospectively defined as partial non-responders between the years 1988 and 2001. Among those were 16 patients for whom the charts contained positive NAB titers (16/568 = 2.8 %). Since we did not carry out systematic antibody testing in all 33 PSTF patients, the percentage of MHDA-positive patients within the PSTF group (48.5 %) is probably underestimated.

### Detection of PSTF

TSUI scores usually decrease after onset of BoNT therapy and continuously improve with repetitive injections every 3 months as demonstrated for a single subject in Fig. 1 (open circle). This patient continuously improved while being injected in our clinic seven times.

As mentioned in the introduction, a systematic shortening of efficacy duration usually provides a first hint for the development of PSTF. In most cases, a reduction of maximal efficacy at week 4 is observed later on in the treatment (Dressler 2004). In our outpatient clinic, patients are injected every 3 months, the duration of efficacy can thus not be monitored directly. However, a systematic worsening of CD severity corresponds to the reduction of duration of efficacy as long as the duration of efficacy is <3 months. Therefore, the charts of 568 CD patients were screened for the presence of a systematic worsening of TSUI scores over two treatment cycles. Two examples are given in Fig. 1. In the first case (dark circle), PSTF occurred very early in the course of BoNT/A treatment 9–12 months after an initial good treatment response. In the other patient, PSTF developed after 38 injections (gray circle).

### Probability of continuous BoNT/A treatment without development of PSTF

**Fig. 2:**
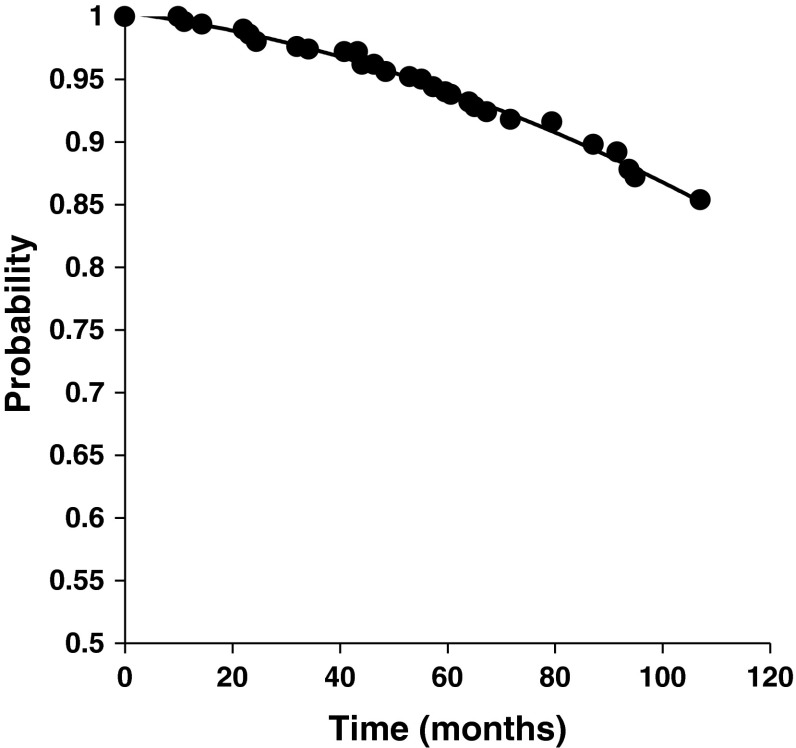
Probability of continuous BoNT/A treatment without the development of partial secondary treatment failure in patients with cervical dystonia (Kaplan–Meier analysis). In case of treatment interruption for at least one treatment cycle (implying that the patient had not been treated for half a year), data were censored from further analysis. An “event” (=occurrence of PSTF) was defined to have occurred at the time when the TSUI score started to worsen systematically (see *arrows* in Fig. 1). For sake of resolution only probabilities between 0.5 and 1.0 are displayed (see* ordinate scale*)

According to our definition of PSTF, a few of our patients already developed PSTF early in the course of BoNT/A treatment. However, there was no clear-cut clustering of PSTF; onset of PSTF seemed to occur over the entire time span analyzed (Fig. 2). The lower frequency of “events” in later months merely reflects the decreasing number of CD patients being continuously treated for a longer time.

The calculation of the Kaplan–Meier plot with censoring of patients with interrupted or ceased treatment for whatever reason resulted in a much higher probability for the occurrence of PSTF than the simple rate of patients with PSTF (*n* = 33) as percentage (5.8 %) of all patients analyzed (*n* = 568). When the occurrence of PSTF at a certain time point was weighted in relation to the number of patients being continuously treated for the entire time span up to that time point, a much higher PSTF probability under continuous BoNT/A treatment was determined. This probability is threefold higher and is 14.5 % over a time span of 108 months. Thus the mean incidence of PSTF per year was 1.61 (= 14.5/108 × 12) %. In contrast to the hypothesis that PSTF mainly occurs early in the course of treatment there was a clear tendency to an increase of PSTF with duration of treatment (see regression parable in Fig. 2).

### Clinical evidence for early efficacy reduction in patients subsequently developing PSTF

**Fig. 3:**
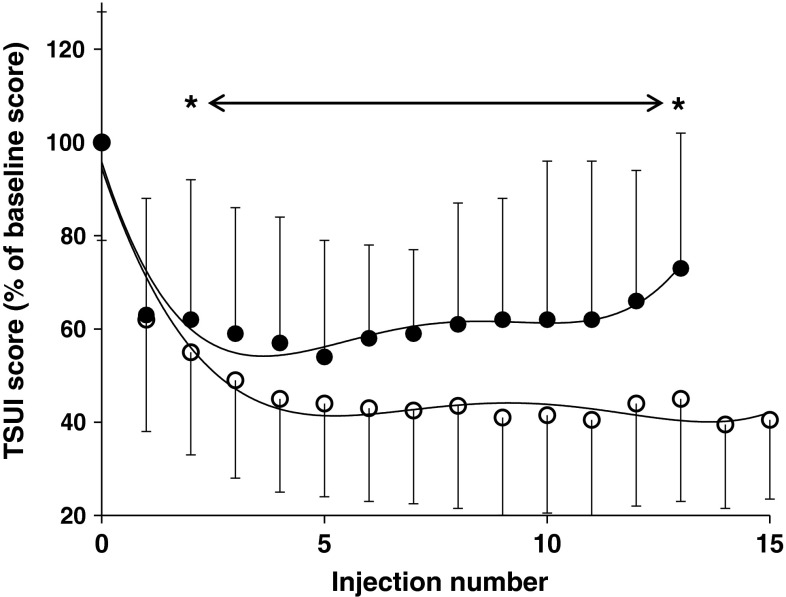
Comparison of standardized mean TSUI scores (± SD) in the PSTF subgroup (*dark circle*; *n* = 33) and the NSTF subgroup (*open circle*; *n* = 535) from the first injection onwards. The first injection had an equal effect in both subgroups. However, mean standardized TSUI scores improved significantly more in the NSTF subgroup than in the PSTF subgroup after the second BoNT/A injection (*asterisk* = *p* < 0.05). The number of patients decreased with number of injections (NSTF subgroup: baseline: *N* = 535; 5 inject.: *N* = 497; 10 inject.: *N* = 429; 15 inject.: *N* = 372; PSTF subgroup: baseline: *N* = 33; 5 inject.: *N* = 32; 10 inject.: *N* = 30; 15 inject.: *N* = 29)

To compare the efficacy of BoNT/A treatment from the very beginning between the NSTF and PSTF subgroup, standardized TSUI scores were calculated for each patient and each injection in both subgroups. The standardized TSUI scores of the PSTF subgroup differed significantly from the standardized TSUI scores of the NSTF subgroup from the third visit onwards (see asterisk in Fig. 3) which took place 3 months after the second injection just before the third injection (Fig. 3). All 33 PSTF patients had an initial good response (and thus were no primary non-responders). The effect of the first injection (controlled at visit 2) was exactly the same in the PSTF and the NSTF subgroup (see Fig. 3).

Mean doses of abobotulinumtoxinA used in the treatment of the PSTF subgroup (752 ± 32 U) were significantly higher than the mean doses of the NSTF subgroup (703 ± 56 U; *p* < 0.01)). In general, the doses used for treatment of CD patients in our clinic between 1988 and 2001 were much higher than the doses used nowadays.

## Discussion

### Early reduction of efficacy in CD patients with subsequent PSTF

Secondary treatment failure to BoNT injections was observed soon after the introduction of BoNT/A in clinical practice (Greene and Fahn 1992; Greene et al. 1994). There is no doubt that BoNT/A resistance may occur very early in the course of treatment even after only a few injections (Dressler and Hallett 2006). This was not only observed in CD treatment but also during therapy for bladder dysfunction (Schulte-Baukloh et al. 2007). Our comparison of a subgroup of patients developing PSTF later on with patients without PSTF showed significant differences in treatment response already after the second BoNT/A treatment and well before patients developed clinically manifest partial/complete secondary treatment failure.

In contrast, clearly delayed onset of resistance to BoNT therapy has also been reported (Tsui et al. 1986; Duane et al. 1995; Dressler and Hallett 2006). Because the number of patients being continuously treated long-term decreases with time, the probability to detect patients with PSTF necessarily also decreases with time. Though STF is described to occur more likely during the first 2–3 years of treatment (Dressler and Hallett 2006), our data demonstrate that the occurrence of PSTF after 4 years is not rare. The decrease of the Kaplan–Meier plot in Fig. 2 suggests that the probability to be treated with BoNT without development of PSTF declines in parallel with the number of patients being treated for a certain time span or even faster.

In some patients, onset of PSTF was detected more than 5 years after onset of BoNT/A therapy. Nevertheless, long before, patient and treating physician realized that a secondary treatment failure had developed, the clinical effect already was significantly less pronounced than in patients not developing PSTF. This is demonstrated in Fig. 3. Therefore, the prevalence of factors leading to STF as for example neutralizing antibodies is probably higher than usually suspected. Whether this prevalence is as high as 40 % as reported by Kranz et al. (2008), has to be analyzed in subsequent studies or falsified by adequate reanalysis of already performed studies.

### PSTF prevalence and incidence in long-term CD treatment

At first glance, the PSTF prevalence rate of 5.8 % in our CD cohort does not seem higher than the rates of secondary non-responders in other larger CD study populations (Zuber et al. 1993; Kessler et al. 1999; Dressler and Hallett 2006; Brin et al. 2008; Naumann et al. 2013). However, the latter rates cannot be used to determine how many patients will develop PSTF during continuous long-term BoNT treatment, because continuity and duration of treatment were not taken into account when calculating these rates. Kaplan–Meier analysis is a more appropriate tool. Because all patients who were not treated continuously are censored, the probability to “survive continuous BoNT treatment without development of PSTF” must necessarily be much higher. Therefore, the estimated rate of PSTF for our CD patients is about threefold higher (14.5 % over a period of 108 months [= 9 years]). The simple calculation of the proportion of patients with PSTF (in a cohort) thus greatly underestimates the incidence of PSTF and therewith the prevalence of PSTF in patients being treated for a certain time span.

This is also true for the treatment with onabotulinumtoxinA. Regarding underestimation of incidence and prevalence there is no difference between the different BoNT/A preparations. However, the individual rates reported in the literature for the different preparations are different (for a review see Naumann et al. 2013).

A statistically more adequate method to estimate PSTF and NAB prevalence was used by Kranz et al. (2008). They randomly selected a small representative sample from their CD cohort still responding to BoNT treatment for MHDA and sweat test and showed a surprisingly high (up to 40 %) PSTF and antibody rate (but they included patients with “borderline” antibody values in the mouse hemidiaphragm test, low antibody titers and continued clinical responsiveness). This may reflect not only differences between centers but might also relate to the reliability of their procedure. Analysis of a second small sample would have reduced the uncertainty of this approach considerably. Furthermore, the sweat test might be more sensitive in detecting PSTF than our method of subsequent scoring of CD severity. In our opinion, the method used by Kranz et al. (2008) is indeed adequate to estimate PSTF prevalence but heavily depends on an appropriate representative sample selection.

### Prevalence of neutralizing antibodies in long-term CD treatment

Interestingly, not all patients with PSTF and STF develop NABs. Greene et al. already reported in 1994 that some patients who had improved after BoNT injections lost efficacy without serological evidence of antibodies. In another study, the secondary non-responder rate was 5 % but only 2 % of 303 CD patients had NABs (Kessler et al. 1999). Voller et al. (2004) reported 18 patients with secondary non-response but only 9 of them had positive NAB titers, and Cordivari et al. (2006) found that not all of the 11 reported patients with poor response to EMG-guided injections had positive antibody titers. In a larger series of blood samples of secondary non-responders (being sent to a central lab) only 44.5 % of the samples were MHDA-positive (Lange et al. 2009). These observations and numbers concur with our data of 16 patients with NABs (= 45.8 %) among 33 PSTF patients. However, according to our Kaplan–Meier PSTF analysis, this implies that NAB prevalence in our CD patients who were continuously treated for 9 years will be at least 6.6 %. Thus, Greene et al. (1994) were correctly guessing that the actual prevalence of serologically detectable antibodies may be higher than 4 %, and the data reported by Kranz et al. (2008) indicate a much higher, probably too high NAB prevalence.

### Implications of high prevalence and early detection of PSTF in long-term CD treatment

Usually (complete) secondary treatment failure is assumed to be present when patient and treating physician do not see a clinical response. However, as pointed out by Dressler (2004), the lack of response does not occur suddenly from one treatment to the next but develops over several treatment cycles starting with a reduction in duration of clinical effect and ending with an additional significant reduction of maximal effect at weeks 2–4. A careful analysis of the clinical effect every 12 weeks (as performed in the present study) is a sensitive tool to detect the systematic reduction of the duration of the clinical effect by documenting a systematic worsening of subsequent TSUI scores. Thus, by means of our definition of PSTF onset of PSTF can be detected early, long before complete secondary therapy failure is observed. Keeping the duration of treatment cycles constant may thus be helpful in detecting PSTF early.

The steepness of a Kaplan–Meier curve very much depends on the definition of PSTF and the sensitivity of measurement. PSTF was detected in some of our patients only by retrospective analysis of their charts but not in clinical routine which indicates that the detection sensitivity may be lower in daily practice as compared to a rigorous retrospective analysis. These considerations imply that it will be difficult to detect PSTF onset in a practice setting where injections are given by different physicians. When patients are scored carefully and the scores are available for each injection session there might be a chance to detect PSTF early.

In summary, evidence is presented here that reduction of efficacy may occur early in the course of BoNT/A treatment in CD patients developing clinical manifest PSTF years later. Careful monitoring and scoring of treatment effect in CD is therefore recommended to detect PSTF early and to avoid the development of complete therapy failure and the induction of high titers of neutralizing antibodies.

## Abbreviations

BoNT/A: Botulinum neurotoxin type A
BoNT/B: Botulinum neurotoxin type B
CD: Cervical dystonia
MHDA: Mouse hemidiaphragm assay
MLA: Mouse lethality assay
NAB: Neutralizing antibody
PSTF: Partial secondary treatment failure
STF: Secondary treatment failure
